# Surgical Treatment after Failed Primary Correction of Urogenital Sinus in Female Patients with Virilizing Congenital Adrenal Hyperplasia: Are Good Results Possible?

**DOI:** 10.3389/fped.2016.00118

**Published:** 2016-10-27

**Authors:** Maria Helena Palma Sircili, Tania Sartori Sanchez Bachega, Guiomar Madureira, Larissa Gomes, Berenice Bilharinho Mendonca, Francisco Tibor Dénes

**Affiliations:** ^1^Division of Urology, Hospital das Clínicas, School of Medicine, University of São Paulo, São Paulo, Brazil; ^2^Division of Endocrinology, Hospital das Clínicas, School of Medicine, University of São Paulo, São Paulo, Brazil

**Keywords:** congenital adrenal hyperplasia, failed feminizing genitoplasty, persistent urogenital sinus, reoperation, follow-up

## Abstract

**Purpose:**

Genital reconstruction in female patients with virilizing congenital adrenal hyperplasia (CAH) is very challenging. Our aim was to evaluate the techniques employed to treat complications after failure of primary urogenital sinus (UGS) surgery, as well as the result of these reoperations.

**Patients and methods:**

Twenty girls with virilizing CAH who were previously submitted to genitoplasty in our service and elsewhere had recurrent UGS stenosis and vaginal introitus stenosis that required surgical treatment. The main symptoms were recurrent urinary tract infection (UTI) in nine, dyspareunia in six, and hematocolpos in three (two associated with sepsis). The anatomical findings were the persistence of UGS with stenosis in 17 patients and vaginal introitus stenosis in 3. The mean age at procedure was 15.2 years, averaging 13.1 years after the first surgery. The surgical techniques employed were isolated perineal flap in 17 patients and perineal flap with partial mobilization of UGS in 3. The mean follow-up after the procedure was 4.8 years (varying from 1 to 17 years).

**Results:**

Vaginal dilations were performed after surgery in 15 patients. Good functional and anatomical results were obtained in 15 patients, with vaginal introitus amenable to dilators of 3.0 cm in diameter. Five patients with high vaginal insertion had recurrent vaginal stenosis and required a surgical revision. No patients presented menstrual obstruction or UTI after surgery. Eight of the 15 adult patients are sexually active.

**Conclusion:**

The reoperation to treat failed primary UGS treatment using Y-V flap and partial mobilization techniques associated with vaginal dilations, promoted good anatomical, and functional results with low morbidity in 75% of the patients.

## Introduction

Congenital adrenal hyperplasia (CAH) is the most frequent cause of disorder of sexual development (DSD). It is associated with abnormally low cortisol and high production of androgens precursors. Increased androgen levels cause *in utero* virilization of female fetus, which at birth presents with clitoral enlargement, urogenital sinus (UGS), and labioscrotal enlargement and fusion (1). In clinical practice, the intensity of external genital virilization is classified into scores that vary from 1 to 5 (2). The challenge of the surgical management comprises patients with Prader scores 3–5 and those with UGS and high vaginal confluence. Urogenital sinus abnormalities occur in a wide spectrum and require different operative procedures to expose individualized urethral and vaginal orifices. These are particularly difficult when the vaginal confluence is high, near the bladder neck, as such patients can remain with persistent UGS symptoms, either in childhood [such as urinary tract infections (UTIs) and post-void dribbling], as well as after puberty (like hematocolpos with or without sepsis), or in adulthood (dyspareunia or incapacity to have sexual intercourse).

The aim of the genital reconstruction in girls with virilizing CAH is to obtain normal female external genitalia with preservation of an innervated, vascularized, but proportional clitoris, as well as to expose the UGS and separate the vaginal from the urethral openings. This is the most important aspect, as it provides a urethra with normal caliber and urinary flow and a vaginal introitus that allows normal menstruation and satisfactory penetration during sexual intercourse in adulthood.

Several techniques were proposed to achieve the best anatomical and functional results. Fortunoff et al. described, in 1964, the perineal flap to separate the vaginal introitus from the urethra, while Hendren and Crawford described the pull-through vaginoplasty in 1969. Passerini-Gazel proposed the use of prepucial flap to pave the distal vagina in 1989, while Gonzalez and Fernandez, in 1990, described the use of prepucial flap to pave the distal anterior vagina, in association with the perineal flap described previously by Fortunoff. In 1997, Peña proposed the total mobilization of the UGS in patients with cloacal malformation, while in 2006, Rink et al. described the partial mobilization of the UGS in CAH patients (3–8).

Congenital adrenal hyperplasia is a lifelong disorder, which poses management problems that are age and sex specific (9, 10). Few data report the long-term results of feminizing genitoplasty and its complications after puberty and adult life (11). There is also lack of information about the need for reoperation as well as of the techniques used for such reconstruction. There are no reports in literature describing surgical techniques to treat failed primary UGS treatment. In this study, we reviewed the techniques used for the reoperation of CAH patients with previous unsuccessful surgical treatment of the USG and their long-term follow-up.

## Patients and Methods

During the period of 1974–2014, 60 patients with virilizing CAH were submitted to feminizing genitoplasty with the isolated Y-V flap technique in our service and three developed complications requiring reoperation to treat them. These 3 and 17 further patients who had their first feminizing genitoplasty performed elsewhere and sought treatment in our service due to complications were included in our study. The mean age at first surgery was 2.1 years, and two patients (cases 16 and 18) had more than one procedure elsewhere to treat UGS before our evaluation. Surgical complications occurred immediately postoperative or up to 28 years after surgery.

The data of the 20 patients were retrospectively reviewed, but the descriptions of the first surgical techniques performed elsewhere were unavailable. Nineteen patients had CAH due to 21-hydroxylase deficiency, 14 of them being salt wasters, while 1 patient due to 11-hydroxylase deficiency. The clinical symptoms were recurrent UTIs in nine patients, hematocolpos in three (two of them associated with sepsis), dyspareunia in six adult patients, and introitus stenosis that precluded penetration at the beginning of sexual activity in three. Physical examination revealed UGS stenosis in 17 patients as well as vaginal introitus stenosis in 3. Associated recurrent clitoromegaly was observed in three.

All patients and parents of under-aged patients signed the informed consent to participate of this study.

In our service, the procedure was performed at a mean age of 15.2 years, representing an average of 13.1 years (ranging from 1 to 28 years) after the first surgery (1987–2015).

At surgery, preliminary cystoscopic evaluation revealed low vaginal insertion in 13 patients and high vaginal insertion in 7 patients. At this moment, balloon catheters were inserted in the bladder and vagina.

All patients with low vaginal confluence and four with high vaginal confluence were initially submitted to Y-V perineal flap, with an inverted Y shaped incision made below the UGS orifice. The flap is developed, taking care to avoid injury of the underlying rectum. The UGS is incised in the midline along its posterior wall up to the urethrovaginal confluence. The incision is then extended 1–2 cm into the vagina wall, in order to insert the apex of the inverted “V” shaped perineal flap into the vaginal introitus, therefore obtaining adequate introitus enlargement.

**Figure 1:**
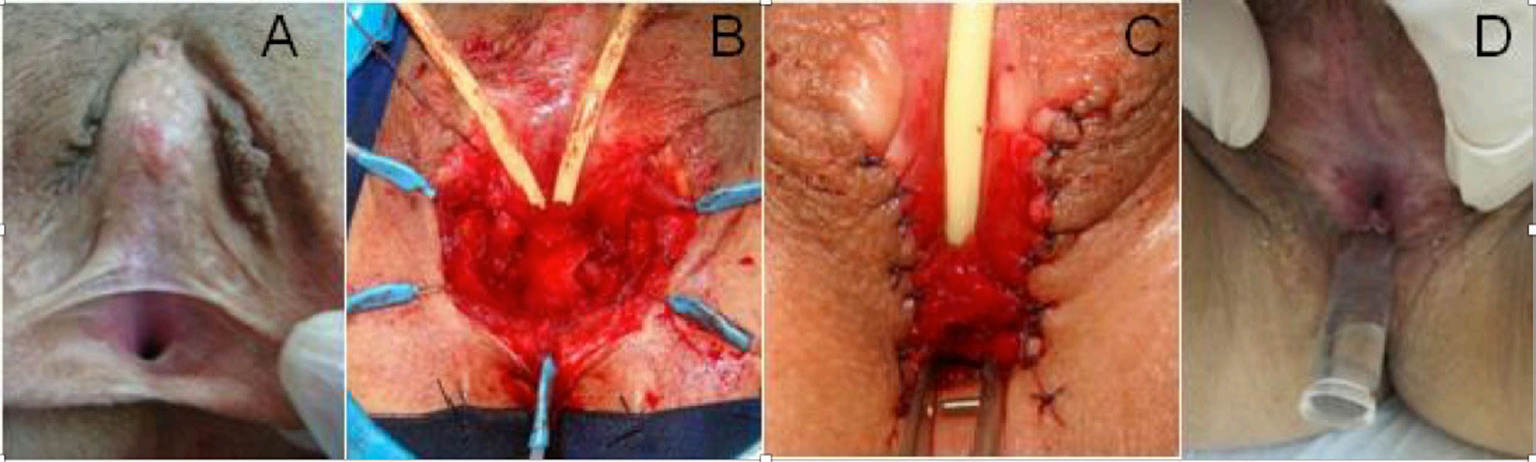
**Patient submitted to previous genitoplasty**. **(A)** Initial aspect with one small perineal orifice, **(B)** perineal flap incision plus partial UGS mobilization, with catheters in the bladder and vagina, **(C)** final surgical aspect with separate orifices for urethra and vagina, and **(D)** late aspect with insertion of vaginal mold.

Partial mobilization of the UGS was employed more recently in three patients with high vaginal insertion. The inverted perineal Y incision is made, without opening the UGS. The lateral and posterior wall of the UGS and vagina are submitted to extensive sharp and blunt dissection, oriented by the balloon catheter inserted in the vagina. After adequate mobilization of the posterior and lateral vaginal walls, a posterior longitudinal incision is made in the UGS until the distal vagina is reached, allowing tension-free suture of its posterior wall to the perineal flap (Figure 1). Three patients with recurrent clitoromegaly were also submitted to concomitant clitoroplasty.

In five patients with unsatisfactory results, another surgical procedure was required, consisting of isolated Y-V perineal flap in three patients, Y-V perineal flap associated with excision of fibrotic tissue in one patient, and Y-V perineal flap with partial UGS mobilization and interposition of buccal mucosa graft due to the lack of hair-free perineal tissue in one patient (Patient 8).

Vaginal dilations to enlarge the introitus are usually indicated when necessary before the initiation of sexual activity in adult patients (12). In our group of adult patients, vaginal dilations were initiated as early as possible after adequate healing of the perineal scars, in order to avoid recurrent vaginal stenosis. In four young patients, aged 10, 12, 14, and 15 years, the dilations were also initiated after the reoperation, under psychological support. Hyaluronidase and betametasone valerate 2.5 mg lubricants were also employed during dilations in patients with intense fibrous tissue.

The median age at the final surgical intervention was 15.5 years (7–31 years). The median patient’s age at the last evaluation was 25.5 years (12–44 years). All patients are now in postpubertal age, but five are younger than 18 years. The median follow-up after the final surgery was 5.2 years (1–17 years). Surgical anatomical results were classified according to previously published criteria (13). Functional results included urinary symptoms, menstrual flow, and sexual activity. Clinical and surgical data are summarized in Table 1.

**Table 1:** **Clinical data, surgical procedures, and final results of the 20 patients**.

Pt	Symptoms	Physical findings	Age 1st reop (yrs)	Technique 1st reop	Age 2nd reop (yrs)	Technique 2nd reop (yrs)	Age last FU	Separate orifices	Sexual activity
1	UTI	UGSS	7	YV-PF			17	Yes	No
2	None	UGSS	17	YV-PF			20	Yes	No
3^a^	Menuria	UGSS	8	YV-PF	18	YV-PF	23	No	Yes
4	UTI	UGSS	15	YV-PF			18	Yes	No
5	None	UGSS	13	YV-PF			22	No	Yes
6	UTI + menuria	UGSS	19	YV-PF	21	YV-PF	22	No	No
7	Menuria	UGSS	24	YV-PF			27	Yes	Yes
8^a^	UTI + VUR + hematocolpos	UGSS + VIS	5	YV-PF	10	PMUGS + BM	12	Yes	No
9	Dyspareunia	VIS	31	YV-PF			36	Yes	Yes
10	None	UGSS	24	YV-PF			26	Yes	No
11	UTI + dyspareunia	UGSS	20	YV-PF			25	Yes	Yes
12	Pollakisuria	UGSS	6	YV-PF	10	YV-PF	24	No	No
13^a^	Hematocolpos	UGSS	10	YV-PF	15	YV-PF	22	Yes	No
14	Dyspareunia	VIS	14	YV-PF			26	Yes	Yes
15	Dyspareunia	UGSS	18	YV-PF			21	Yes	Yes
16^a^	Dyspareunia + urine incontinence	UGSS	27	YV-PF			44	No	Yes
17	None	UGSS	21	YV-PF			22	Yes	No
18^a^	UTI + hematocolpos	VIS	12	YV-PF + PMUGS			13	Yes	No
19^a^	None	UGSS	14	YV-PF + PMUGS			15	Yes	No
20^a^	UTI	UGSS	15	YV-PF + PMUGS			16	Yes	No

*Pt, patient; Reop, reoperation; yrs, years; FU, follow-up; UTI, urinary tract infection; VUR, vesico-ureteral reflux; UGSS, urogenital sinus stenosis; VIS, vaginal introitus stenosis; Y-VPF, “Y-V” perineal flap; PMUGS, partial mobilization of the urogenital sinus; BM, buccal mucosa*.

*^a^Patients with high vaginal confluence*.

## Results

Reoperation using the Y-V perineal flap was performed in 17 patients. In these, separate orifices for urethra and vagina were obtained in 10 patients (being considered as good results), while 7 patients remained with persistent, albeit shorter UGS with a sole perineal orifice (considered as regular results). From the latter group, two patients were successfully treated only with UGS dilation, while five patients required a third surgical intervention. After this procedure, two patients achieved separate orifices for urethra and vagina, while the other three had to be submitted to dilations in order to maintain their sole orifice enlarged, remaining without complications. The patient who underwent labial mucosa graft had an excellent postoperative course, with good uptake of the graft and exposure of both urethral and vaginal orifices.

Reoperation using Y-V perineal flap associated with partial mobilization of vaginal posterior wall was performed in three patients and separated orifices for urethra and vagina were obtained in all of them. The surgical results are presented in Table 1.

In our group of patients, vaginal dilations were required in 11 adult patients after adequate healing of the perineal scars, in order to avoid recurrent vaginal stenosis. Seven of them referred later normal sexual activity. In four young patients, aged 10, 12, 14, and 15 years, the dilations were initiated under psychological support, successfully precluding recurrent stenosis. Hyaluronidase and betametasone valerate 2.5 mg lubricants were also employed during dilations in patients with intense fibrous tissue.

Considering the total number of patients, final good functional and anatomical results were obtained in 15 patients (75%), with separate orifices and vaginal introitus amenable for dilatation with molds. Five patients (25%) presented anatomically regular results due to a persistent UGS with a sole perineal orifice. Nevertheless, they did not present any urinary symptoms or obstruction of menstrual flow. They were also able to dilate their perineal orifice with molds, while three of them are able to have normal sexual intercourse. Urinary infection occurred in only one of these patients (patient 20) 1 week after surgery.

In total, 8 of the now 15 adult patients are sexually active without dyspareunia.

## Discussion

The treatment of the UGS remains the most challenging aspect in the feminizing genitoplasty in patients with CAH. The separation of the urinary and genital tracts is usually straightforward when the UGS is short, with low vaginal confluence, but can be difficult when UGS is long, with high vaginal confluence (14–16). If this separation is unsuccessful, the UGS may persist or recur while injury to the urethra or vagina may ensue (17).

The incidence of complications of the primary surgery is not well known. Of the 60 patients who underwent primary UGS treatment in our service, with both perineal flap plasty and its association with partial UGS mobilization, only 3 developed complications that required reoperation, representing an incidence of 5%. Nevertheless, the number of our reoperated patients is larger, because being a quaternary center, our service receives patients from distant parts of the country, as well as from neighboring ones.

Several techniques have been proposed to improve the outcomes in UGS with high vaginal insertion, but they are still controversial and may present significant morbidity.

The Y-V perineal flap vaginoplasty is a safe procedure that preserves the urethro-vesical innervations and, therefore, has low risk of urinary incontinence. It is indicated in patients with low urethrovaginal confluence, in which it is easy to individualize the urethra and vaginal orifices inside the UGS (18). This procedure is not indicated in patients with high vaginal insertion as it eventually cannot achieve the complete exteriorization of the UGS, resulting in its persistence, even after reoperation with the same technique. This was observed in five of the primary procedures and in three of the secondary procedures. Although this can sometimes be the cause of urinary symptoms and infection, as well as dyspareunia, three of our patients with persistent UGS in the late follow-up had none of these manifestations.

The perineal flap associated with partial mobilization of the UGS interesting the lateral and posterior vaginal walls provides a good exposure of the high vaginal introitus. This technique avoids dissection of the proximal urethra, preserving the perineal innervations, and the urethral sphincter. There are no reports of incontinence with this technique (19–21). It was employed in three of the first reoperations and one of the second reoperations, totaling four patients with high vaginal insertion, and all of them achieved separate urethral and vaginal orifices in the perineum.

The association of regular dilation helped to stabilize the diameter of the perineal orifices, even in cases with regular surgical result (persistent UGS).

## Conclusion

In our hands, the primary feminizing genitoplasty in girls with CAH has good results in 95% of the cases. In patients who failed primary UGS treatment, secondary treatment produced good results with separate perineal orifices and normal menstrual flow and urination in 75%, with one procedure in 15 patients and two in 5. Surgery with the isolated Y-V perineal flap plasty had satisfactory results in patients with low vaginal insertion, while its association with partial UGS mobilization produced excellent results in all cases. The use of mucosal graft as well as regular dilations and use of hyaluronidase/betametasone valerate lubricants in patients with intense fibrotic tissue helped to avoid introitus stenosis.

## Author Contributions

MS is the surgeon, collected the cases, and wrote the text. TB helped collect the cases and write the text. GM helped collect the cases. LG helped collect the cases. BM is the head of the Department of Endocrinology and helped write the text. FD is the head of the Uropediatric Unit as well as the chief surgeon, helped write, and revised the text.

## Conflict of Interest Statement

The authors declare that the research was conducted in the absence of any commercial or financial relationships that could be construed as a potential conflict of interest.
